# Prevalence of and Changes in Tooth Loss Among Adults Aged ≥50 Years with Selected Chronic Conditions — United States, 1999–2004 and 2011–2016

**DOI:** 10.15585/mmwr.mm6921a1

**Published:** 2020-05-29

**Authors:** Marcia L. Parker, Gina Thornton-Evans, Liang Wei, Susan O. Griffin

**Affiliations:** ^1^Division of Oral Health, National Center for Chronic Disease Prevention and Health Promotion, CDC; ^2^DB Consulting Group, Inc., Atlanta, GA.

Extensive tooth loss can lead to poor diet resulting in weight loss or obesity (*1*). It can also detract from physical appearance and impede speech, factors that can restrict social contact, inhibit intimacy, and lower self-esteem (*1*). Chronic medical conditions and oral conditions share common risk factors (*2*). Persons with chronic conditions are more likely to have untreated dental disease, which can result in tooth loss. Three measures of tooth loss during 1999–2004 and 2011–2016 were estimated by comparing data from the National Health and Nutrition Examination Survey (NHANES) for each period among adults aged ≥50 years with selected chronic conditions.* The three measures were 1) edentulism (having no teeth); 2) severe tooth loss (having eight or fewer teeth) (*3*); and 3) lacking functional dentition (having <20 teeth out of 28, which is considered a full set for the purpose of NHANES assessments) (*4*). During 2011–2016, prevalences of edentulism and severe tooth loss were ≥50% higher among adults with fair or poor general health, rheumatoid arthritis, asthma, diabetes, emphysema, heart disease, liver condition, or stroke than among those with those adults without the chronic condition. Lack of functional dentition was also more prevalent among adults with chronic conditions than among persons without these conditions. Tooth loss is preventable with self-care and routine dental visits (*1*). To encourage these behaviors, public health professionals can educate the public about the association between having a chronic condition and tooth loss, and primary care providers can educate their patients about the importance of healthy behaviors and screen and refer them for needed dental care.

Data obtained from CDC’s NHANES, a multistage probability sample designed to assess the health and nutritional status of the noninstitutionalized U.S. population through participant interviews and physical examinations,^†^ were analyzed for all adults aged ≥50 years and those with selected chronic conditions; the analysis was limited to adults who completed a dental examination as part of NHANES. Prevalences of the three categories of tooth loss (edentulism and severe tooth loss [determined by the Global Burden of Disease to cause major difficulty in eating meat, fruits, and vegetables (*3*)] and lacking functional dentition), using the World Health Organization criteria (*4*) during 2011–2016 were estimated. Lack of functional dentition provides the most actionable information among the three measures because it detects symptomatic tooth loss in the earliest stage. Chronic conditions were self-reported except for uncontrolled diabetes, obesity, and the number of teeth lost, which were clinically assessed. Estimated prevalence of tooth loss and chronic conditions were standardized to the U.S. 2000 Census population by 5-year age increments and sex. T-tests were used to determine whether prevalence of each category of tooth loss varied by chronic disease status and whether lack of functional dentition differed from 1999–2004 to 2011–2016. All analyses were conducted using SAS-callable SUDAAN software (version 11.0.3; RTI International), which accounted for the examination sample weights and the complex, clustered design of NHANES.

The study sample comprised 6,283 adults during 1999–2004 and 7,443 during 2011–2016. During these periods, the following respective prevalences of selected chronic conditions were reported: fair or poor general health (24.5%, 21.7%), any arthritis (43.3%, 45.0%), rheumatoid arthritis (16.3%, 6.1%), asthma (5.4%, 8.9%), diabetes (13.7%, 17.7%), emphysema (4.1%, 3.7%), heart disease (16.7%, 13.4%), liver condition (1.6%, 2.6%), and history of stroke (5.4% during both periods) (Table 1).

**TABLE 1:** Case definitions and prevalences* of selected chronic conditions among adults aged ≥50 years — National Health and Nutrition Examination Survey, United States, 1999–2004 and 2011–2016

Health condition	Case definition	Prevalence, % (SE)	
		1999–2004	2011–2016
General health (fair or poor)	Reporting fair or poor general health versus excellent, very good, or good	24.5 (1.1)	21.7 (1.0)
Any arthritis	Answered “yes” to ever being told had arthritis	43.3 (0.8)	45.0 (0.9)
Rheumatoid arthritis	Answered “yes” to ever being told had arthritis and “yes” to having rheumatoid arthritis	16.3 (0.6)	6.1 (0.4)
Asthma	Answered “yes” to both ever being told had asthma and “yes” to still having asthma	5.4 (0.4)	8.9 (0.6)
Diabetes	Answered “yes” to ever being told had diabetes by doctor or other health care professional	13.7 (0.6)	17.7 (0.6)
Uncontrolled diabetes	Glycohemoglobin level ≥6.5%	11.6 (0.5)	14.6 (0.6)
Emphysema	Answered “yes” to ever being told had emphysema by a doctor or other health care professional	4.1 (0.4)	3.7 (0.3)
Heart disease	Answered “yes” to ever being told had congestive heart failure, coronary heart disease, angina/angina pectoris, or heart attack by a doctor or other health care professional	16.7 (0.8)	13.4 (0.5)
Liver condition	Answered “yes” to both ever being told had any kind of liver condition by a doctor or other health care professional and “yes” to still having a liver condition	1.6 (0.2)	2.6 (0.3)
Obesity	Body mass index score (determined during clinical examination) ≥30 kg/m^2^	32.7 (1.0)	39.9 (1.0)
Stroke	Answered “yes” to ever being told had a stroke	5.4 (0.3)	5.4 (0.3)

**Abbreviation:** SE = standard error.

* All estimates were standardized by using 5-year age increments and sex to U.S. 2000 Census population.

During 2011–2016, among adults who had a dental exam, the prevalences of edentulism, severe tooth loss, and lacking functional dentition were 10.8%, 16.9%, and 31.8%, respectively (Table 2). The prevalences of edentulism and severe tooth loss were higher among persons with each selected chronic condition except obesity than they were among those who did not have the condition. The prevalence of edentulism was at least twice as high among adults with fair or poor general health, emphysema, heart disease, or stroke history as it was among those without the condition; the prevalence of severe tooth loss was ≥50% higher for adults with fair or poor general health, rheumatoid arthritis, asthma, diabetes, uncontrolled diabetes, emphysema, heart disease, liver condition, or stroke, compared with those who did not have the condition.

**TABLE 2:** Prevalences and prevalence ratios* of edentulism, severe tooth loss, and lack of functional dentition among U.S. adults aged ≥50 years with and without selected chronic conditions who had a dental exam — National Health and Nutrition Examination Survey, United States, 2011–2016

Condition	Edentate (zero teeth)		Severe tooth loss (≤8 teeth)		Lack of functional dentition (<20 teeth)	
	% (SE)	Prevalence ratio^†^	% (SE)	Prevalence ratio^†^	% (SE)	Prevalence ratio^†^
**All**	**10.8 (0.8)**	**N/A**	**16.9 (1.0)**	**N/A**	**31.8 (1.2)**	**N/A**
**General health**						
Fair or poor	19.2 (1.7)^§^	2.29	30.2 (1.8)^§^	2.31	52.4 (2.1)^§^	2.01
Good or better	8.4 (0.6)		13.1 (0.8)		26.1 (1.1)	
**Any arthritis**						
Yes	12.3 (1.2)^§^	1.24	18.6 (1.3)^§^	1.18	35.8 (1.7)^§^	1.22
No	9.9 (0.8)		15.7 (1.0)		29.3 (1.1)	
**Rheumatoid arthritis**						
Yes	18.2 (2.6)^§^	1.77	25.1 (2.7)^§^	1.54	48.3 (2.5)^§^	1.57
No	10.3 (0.8)		16.3 (1.0)		30.7 (1.2)	
**Asthma**						
Yes	16.9 (2.0)^§^	1.64	24.9 (2.2)^§^	1.54	44.1 (3.1)^§^	1.44
No	10.3 (0.8)		16.2 (1.0)		30.6 (1.2)	
**Diabetes**						
Yes	15.2 (1.6)^§^	1.52	24.2 (2.0)^§^	1.56	43.4 (2.3)^§^	1.46
No	10.0 (0.8)		15.5 (1.0)		29.7 (1.3)	
**Uncontrolled diabetes**						
Yes	13.8 (1.7)^§^	1.35	23.4 (1.9)^§^	1.51	42.2 (2.2)^§^	1.42
No	10.2 (0.8)		15.5 (1.0)		29.8 (1.2)	
**Emphysema**						
Yes	39.2 (5.6)^§^	3.96	49.1 (6.0)^§^	3.11	66.1 (5.9)^§^	2.15
No	9.9 (0.7)		15.8 (0.9)		30.7 (1.1)	
**Heart disease**						
Yes	20.7 (2.6)^§^	2.11	29.3 (2.2)^§^	1.89	51.2 (3.2)^§^	1.72
No	9.8 (0.7)		15.5 (1.0)		29.8 (1.2)	
**Liver condition**						
Yes	16.2 (2.5)^§^	1.51	26.5 (3.3)^§^	1.60	45.7 (4.5)^§^	1.45
No	10.7 (0.8)		16.6 (1.0)		31.5 (1.2)	
**Obesity**						
Yes	11.9 (1.0)	1.16	18.8 (1.0)^§^	1.19	35.6 (1.5)^§^	1.23
No	10.3 (0.9)		15.8 (1.2)		29.0 (1.4)	
**Stroke**						
Yes	22.6 (3.2)^§^	2.24	35.0 (3.7)^§^	2.20	55.8 (3.4)^§^	1.82
No	10.1 (0.8)		15.9 (1.0)		30.7 (1.2)	

**Abbreviations:** N/A = not applicable; SE = standard error.

* All estimates were standardized by using 5-year age increments and sex to U.S. 2000 Census population.

^†^ Prevalence in group with condition divided by prevalence in those without condition.

^§^ Statistically significant (p<0.05).

The overall prevalence of lack of functional dentition decreased 11.7 percentage points from 1999–2004 (43.5%) to 2011–2016 (31.8%) (Figure) (Supplementary Table, https://stacks.cdc.gov/view/cdc/88330). Improvements were detected for persons with fair or poor general health, any arthritis, diabetes, and obesity. The most notable improvements were among persons reporting diabetes (16.6 percentage-point decrease) and uncontrolled diabetes (18.8 percentage-point decrease). Prevalence of lack of functional dentition increased by 11.2 percentage points among persons with rheumatoid arthritis during this period. During 2011–2016, lack of functional dentition was ≥50% more prevalent among adults reporting fair or poor general health, rheumatoid arthritis, emphysema, or heart disease than among those not reporting the condition (Supplementary Table, https://stacks.cdc.gov/view/cdc/88330).

**FIGURE:**
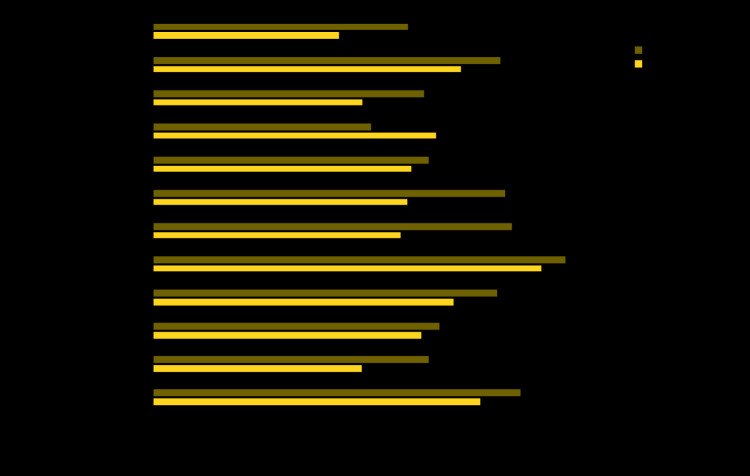
Change in prevalence*^,†^ of lack of functional dentition (<20 teeth) among U.S. adults aged ≥50 years with selected chronic conditions — National Health and Nutrition Examination Survey, United States, 1999–2004 and 2011–2016 * All estimates were standardized to the 2000 U.S. Census population by 5-year age increments and sex. ^†^ Change in prevalence is statistically significant (p<0.05) for all conditions except asthma, emphysema, heart disease, liver condition, and stroke.

## Discussion

Among adults aged ≥50 years who had a dental exam as part of NHANES, having at least one selected chronic condition was associated with increased tooth loss. Studies using earlier NHANES data also found this association (*1*,*2*). Although the prevalence of lack of functional dentition largely decreased from 1999–2004 to 2011–2016, the association between tooth loss and having a chronic condition remained, and among persons who reported having rheumatoid arthritis, the prevalence of lack of functional dentition increased. The reasons for this finding are not known; the prevalence of reported rheumatoid arthritis decreased substantially (>60%) from 1999–2004 to 2011–2016 (Table 1), so the increase in prevalence of lack of functional dentition among persons with rheumatoid arthritis could possibly be attributable to changes in the sample composition between surveys.

Dental caries and periodontal (gum) disease are the leading causes of tooth loss; both are preventable. Primary prevention of caries includes treatment with fluoride applied professionally or at home and added to drinking water; scaling and root planing in a dental office can also prevent and stop the progression of periodontal disease (*1*). In addition to fluoride, dental fillings (restorations) can also prevent the progression of caries. A 2009 analysis of 1999–2004 NHANES data found that after controlling for covariates, (e.g., race/ethnicity and income), persons with chronic conditions had higher levels of unmet dental treatment needs than did persons without chronic conditions; obesity, diabetes, emphysema, and stroke were associated with a higher prevalence of unmet need for treatment of caries, and diabetes and obesity were associated with higher prevalences of unmet need for treatment of periodontitis (*2*).

Because traditional Medicare (Parts A and B) does not cover routine dental care, older adults with chronic conditions might have difficulty accessing clinical dental care because they lack dental insurance. Some Medicare Advantage plans (Part C), however, do cover routine dental services (*5*). Persons with low household income might also lack access because of the limited dental safety net; in 2019, only 18 states and the District of Columbia offered extensive dental services to adults enrolled in Medicaid (*6*). In addition, chronic conditions can limit mobility, which might make visiting a dentist and maintaining good home care more difficult.

Data from the 2017 Medical Expenditure Panel Survey indicated that >40% of adults aged ≥65 years had a past-year visit to a physician’s office but no visit to a dentist (Agency for Healthcare Research and Quality, unpublished analysis, 2019). Better integration and collaboration between all providers could improve health care outcomes. Health care professionals can thus play an important role in helping their patients with chronic conditions keep their natural teeth. Providers can educate these patients about their higher risk for tooth loss and the importance of preventive care administered at home or received in a dental office.

Primary care providers can also screen patients for common dental conditions and refer them for necessary care. A 2011 Institute of Medicine report^§^ found that health care professionals, with proper training, can assess risk and screen for common oral conditions (*7*); an oral health curriculum designed for medical providers is available on the Smiles for Life website (*8*). Nonprofit organizations can also play a role in preventing tooth loss by educating their constituents about their higher risk for tooth lost and need for prevention. Among the chronic conditions included in this review, the only one with recommendations for routine dental visits as the standard of care is diabetes (*9*). A Cochrane review found some evidence that treating periodontitis can improve outcomes (i.e. glycemic control) among persons with diabetes (*10*). In this study, improvements in maintaining functional dentition were notably high among persons with diabetes.

The findings in this report are subject to at least three limitations. First, the data for most chronic conditions were self-reported. Second, the prevalences of some chronic conditions were low; therefore, there might have been insufficient power to detect a significant difference. Small sample size also might have contributed to statistically unreliable changes among persons reporting emphysema, a liver condition, or stroke history. Finally, some covariates associated with chronic disease such as race/ethnicity and income were not controlled for; therefore, differences in dental health status between persons with and without chronic conditions could also have been attributable to these factors.

During 2016–2018, CDC funded programs in six states to enhance understanding of connections between chronic disease and oral health in state health department programs. Several states initiated pilot projects to implement strategies for better coordination of medical and dental care. CDC currently supports medical-dental integration activities to increase bidirectional messaging and referrals for dentists and primary care providers serving patients with prediabetes, diabetes, and hypertension.^¶^ Information obtained from these activities can be used to develop effective approaches to reduce the high prevalence of tooth loss among persons with chronic conditions and support better chronic disease management.

**Summary**

**What is already known about this topic?**

Older adults are more likely to annually visit a doctor than a dentist. Certain chronic conditions are associated with severe tooth loss, which can diminish quality of life and interfere with eating healthy foods.

**What is added by this report?**

Among adults aged ≥50 years who had a dental exam as part of the National Health and Nutrition Examination Survey, those reporting selected chronic conditions were significantly more likely to have severe tooth loss than were persons without chronic conditions.

**What are the implications for public health practice?**

Health care professionals can educate patients with chronic diseases about their increased risk for tooth loss, screen for dental disease, and refer patients for needed dental care.

## Footnotes

* Data from three 2-year cycles of NHANES were combined for each period: 1999–2000, 2001–2002, and 2003–2004 and 2011–2012, 2013–2014, and 2015–2016, respectively.

† https://www.cdc.gov/nchs/nhanes.htm.

§ Currently known as The National Academies of Science, Engineering, and Medicine.

¶ https://www.cdc.gov/chronicdisease/resources/publications/aag/oral-health.htm.
